# Insight into the on/off switch that regulates expression of the MSMEG-3762/63 efflux pump in *Mycobacterium smegmatis*

**DOI:** 10.1038/s41598-023-47695-4

**Published:** 2023-11-21

**Authors:** Nicoletta Campolattano, Gianluca D’Abrosca, Luigi Russo, Barbara De Siena, Milena Della Gala, Ida De Chiara, Rosangela Marasco, Aaron Goff, Simon J. Waddell, Margherita Sacco, Lidia Muscariello

**Affiliations:** 1 Dipartimento di Scienze e Tecnologie Ambientali Biologiche e Farmaceutiche, Università degli Studi della Campania Luigi Vanvitelli, Caserta, Italy; 2 Department of Global Health and Infection, Brighton and Sussex Medical School, University of Sussex, Brighton, BN1 9PX UK; 3 Present Address: Department of Clinical and Experimental Medicine, University of Foggia, Foggia, Italy

**Keywords:** Bacterial genes, Tuberculosis, Antimicrobial resistance

## Abstract

Drug resistance is one of the most difficult challenges facing tuberculosis (TB) control. Drug efflux is among the mechanisms leading to drug resistance. In our previous studies, we partially characterized the ABC-type MSMEG-3762/63 efflux pump in *Mycobacterium smegmatis*, which shares high percentage of identity with the *Mycobacterium tuberculosis* Rv1687/86c pump. MSMEG-3762/63 was shown to have extrusion activity for rifampicin and ciprofloxacin, used in first and second-line anti-TB treatments. Moreover, we described the functional role of the TetR-like MSMEG-3765 protein as a repressor of the *MSMEG_3762/63/65* operon and orthologous *Rv1687/86/85c* in *M. tuberculosis*. Here we show that the operon is upregulated in the macrophage environment, supporting a previous observation of induction triggered by acid-nitrosative stress. Expression of the efflux pump was also induced by sub-inhibitory concentrations of rifampicin or ciprofloxacin. Both these drugs also prevented the binding of the MSMEG-3765 TetR repressor protein to its operator in the *MSMEG_3762/63/65* operon. The hypothesis that these two drugs might be responsible for the induction of the efflux pump operon was assessed by bioinformatics analyses. Docking studies using a structural model of the regulator MSMEG-3765 showed that both antibiotics abolished the ability of this transcriptional repressor to recognize the efflux pump operon by interacting with the homodimer at different binding sites within the same binding pocket. Reduced binding of the repressor leads to induction of the efflux pump in *M. smegmatis*, and reduced efficacy of these two anti-mycobacterial drugs.

## Introduction

*Mycobacterium tuberculosis* causes tuberculosis (TB), an airborne transmissible human infection, one of the major causes of death worldwide^1^. An increase in TB deaths was registered for the first time in the last 10 years, raising from 1.4 in 2019 to 1.5 million in 2020, probably due to reduced access to early TB diagnosis and treatment during the SARS-CoV-2/COVID-19 pandemic. It has been estimated that half of the people affected by TB did not have access to care, and that about 16% of people ill with multi-drug resistant TB (MDR-TB) did not receive adequate treatment during the SARS-CoV-2/COVID-19 pandemic^1,2^. The continued emergence of MDR clinical isolates of *M. tuberculosis*, as well as of other important human pathogens, represents a public health alert worldwide^3–6^. Drug resistance is usually conferred by mutations in genes for target proteins or in genes responsible for drug activation^3,7–10^. Nevertheless, a series of physiological events have been found in clinical isolates of many human pathogens occurring at different stages of infection where the phenotypic state of the bacteria may reduce drug efficacy^11–14^. Indeed, *M. tuberculosis* populations may develop a temporary ability to survive in the absence of resistance-conferring mutations, leading to the phenomenon of drug tolerance, which may partly account for the heterogeneous populations of bacteria found in patients^12^. Drug tolerance is due to different mechanisms, including decreased growth rate, shifted metabolic states and increased expression of efflux pumps^15,16^. Moreover, increased drug efflux, acting synergistically with the low permeability of the mycobacterial cell wall, occurring as an early stress response to drug exposure, may constitute an early step in the development of stable and heritable drug resistance^12,17,18^. In mycobacteria, efflux pumps that belong to the ABC (ATP binding cassette) and MFS (major facilitator) families are involved in drug efflux^19^.

We have previously characterized the ABC-type MSMEG-3762/63 efflux pump in *Mycobacterium smegmatis*^20,21^, a mycobacterium widely used as a model system for *M. tuberculosis* physiology^22,23^. This efflux pump shares a high percentage identity (68% and 79%, for The ATP binding protein and the ABC transporter, respectively) with the *M. tuberculosis* Rv1687/86c pump^20^. In our previous studies, we demonstrated that the TetR-like MSMEG-3765 repressor was able to negatively regulate the expression of the *MSMEG_3762/63/65* operon and of the homologous *Rv1687/86/85c* operon, encoding the efflux pump and its regulator Rv1685c in *M. tuberculosis*^20^. We also showed that the MSMEG-3762/63 efflux pump was involved in the extrusion of rifampicin and ciprofloxacin in *M. smegmatis* by comparative analysis of wild-type and *M. smegmatis (∆_MSMEG_3763*) null mutant strains^21^. Here, measuring the transcriptional regulation of the *MSMEG_3762/63/65* efflux pump operon, we provide evidence that the operon is induced after challenge with sub-inhibitory concentrations of rifampicin and ciprofloxacin, as well as in bacilli within macrophages. Moreover, by combining structural modeling methodologies with molecular docking techniques, we report the structural determinants governing the DNA and ligand recognition mechanisms of the TetR-like MSMEG-3765 homodimer, and show that the presence of these anti-TB drugs abrogates repressor binding leading to induction of the pump and enhanced drug efflux in *M. smegmatis*.

## Results

### Absence of the MSMEG-3762/63 pump enhances membrane potential

**Figure 1:**
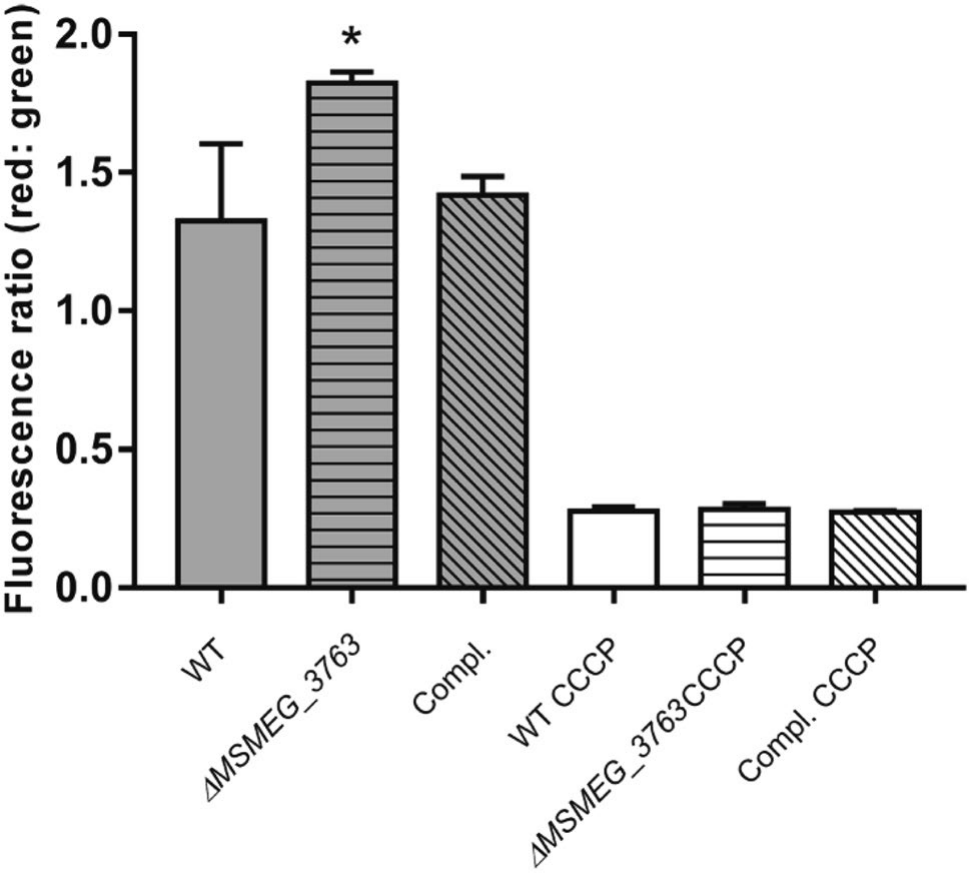
Comparative analysis of membrane potential. Comparison of the Δψ in *M. smegmatis* (wt), *M. smegmatis* (*ΔMSMEG_3763*) and complemented *M. smegmatis* (*ΔMSMEG_3763* pBD04) (Compl.) was performed using the DiOC_2_ fluorescent dye and the ratiometric method (Red:Green). Data are the average of biological and technical triplicates in the absence and presence of the depolarizing compound CCCP. Error bars represent the standard deviation of the mean values. Significance of data obtained was tested by the Student’s t test (^∗^*p* < 0.05, compared to wt and Compl.).

To evaluate the functional role of MSMEG-3762/63 in membrane potential (Δψ), cytofluorimetric analysis was performed on *M. smegmatis* (wt), *M. smegmatis* (*ΔMSMEG_3763*) and *M. smegmatis* (*ΔMSMEG_3763* pBD04) complemented strain, using the DiOC_2_ fluorescent carbocyanine dye. DiOC_2_ exhibits green fluorescence in the monomeric state but shifts toward red emission as the dye molecules form aggregates at high cytosolic concentrations caused by higher membrane potentials; the green–red shift disappears in the presence of the depolarizing compound CCCP. Comparative analysis of the three strains showed that *ΔMSMEG_3763* had a greater membrane potential than the wt strain (Fig. 1), and that the wt phenotype was restored in the complemented strain. The higher membrane potential in the *ΔMSMEG_3763* null mutant suggests that the pump may be involved in both drug and ion transport, as described for other ABC pumps^24,25^.

### Phenotypic analysis for drug-sensitivity in the *M. smegmatis* (∆*MSMEG_3763*) mutant strain

The increased efficacy of rifampicin and ciprofloxacin in null mutants of the MSMEG-3762/63 efflux pump was previously established^21^. To extend these findings to other first- and second-line anti-TB drugs, minimum inhibitory concentrations (MICs) were determined using the Resazurin Microtiter Assay (REMA). *M. smegmatis* mc^2^ 155 (wt) and its isogenic deletion mutant (*ΔMSMEG_3763*) and complemented (*ΔMSMEG_3763* pBD04) strains were grown to log phase and exposed to increasing concentrations of first-line (isoniazid, ethambutol, streptomycin) and second-line (levofloxacin, moxifloxacin, gatifloxacin) anti-TB drugs (Supplementary Table S2). In contrast to rifampicin and ciprofloxacin, no variations in MIC values of the null mutant compared to wt and complemented strains were detected, indicating that the MSMEG-3762/63 pump is not involved in the extrusion of these antibiotics. Therefore, we focused on the role of MSMEG-3762/63 in the efflux of rifampicin and ciprofloxacin.

### The *MSMEG_3762/63/65* operon is induced on exposure to sub-inhibitory antimicrobial drug concentrations

**Figure 2:**
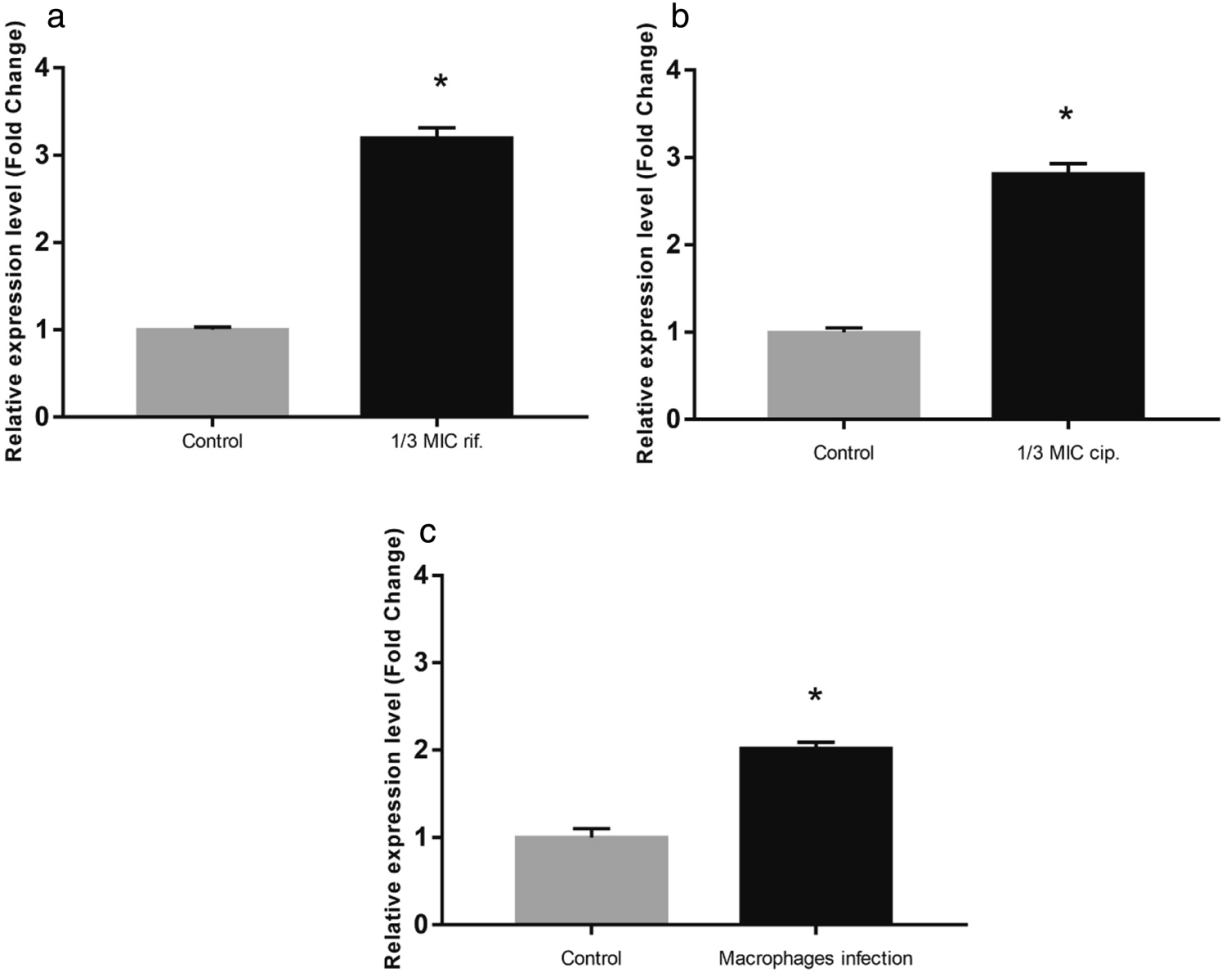
Transcriptional analysis in stress conditions. Comparative RT-qPCR analysis showing induction of *the MSMEG_3762/63/65* operon in log phase culture after exposure to 1/3^rd^ MIC rifampicin (**a**) and 1/3^rd^ MIC ciprofloxacin (**b**) or after macrophage phagocytosis (**c**). Control in (**a**) and (**b**): cells grown in the absence of antibiotics; control in (**c**): cells grown in RPMI in the absence of macrophages. *sigA* was used as a reference gene and relative expression levels were calculated with the Pfaffl method (**a**,**b**) or the 2^−ΔΔCt^ method (**c**). Data shown are the mean values of three independent experiments, and each RT-qPCR was carried out in technical triplicate. Error bars represent the standard deviation of the mean values. Significance of data obtained was tested by the Student’s t test (^∗^*p* < 0.05).

To investigate the possible role of rifampicin and ciprofloxacin as transcriptional inducers of the *MSMEG_3762/63/65* operon, RT-qPCR analyses were performed on *M. smegmatis* wt cells exposed to sub-inhibitory concentrations (1/3rd MIC) of rifampicin (MIC = 3 μg mL^−1^) and ciprofloxacin (MIC = 0.25 μg mL^−1^). The expression of the operon increased 3.2-fold in the presence of rifampicin (Fig. 2a) and 2.8-fold in the presence of ciprofloxacin (Fig. 2b) compared to drug-free control bacilli. These data suggest that rifampicin and ciprofloxacin induce expression of this efflux pump system by binding the MSMEG-3765 TetR-like transcriptional regulator that controls expression of the efflux pump.

### The efflux pump operon *MSMEG_3762/63/65* is overexpressed in macrophages

In order to investigate the putative involvement of the efflux pump in the resistance to stress encountered within macrophage environment, the expression level of the efflux pump operon in macrophage was analysed. For this aim, human THP-1 monocytes were differentiated into macrophages and exposed to *M. smegmatis* (MOI 10:1). Phagocytosis was permitted for 2 h, after which the macrophages were lysed and RNA was extracted from the intracellular mycobacteria. The expression of the *MSMEG_3762/63/65* operon was twofold higher compared to cells grown in RPMI alone (Fig. 2c). These results are consistent with the finding of Cossu and co-worker^26^. They reported that the mRNA profiles of *M. tuberculosis* and *M. smegmatis* exposed to acid-nitrosative stress, mimicking the macrophage environment, showed a high number of differentially expressed genes. In particular, the *MSMEG_3762/63/65* operon was upregulated, and this observation was confirmed by GFP promoter probe analysis^20^. All together these data support the hypothesis that the MSMEG-3762/63 efflux pump, and likely homologous Rv1687/86c in *M. tuberculosis*, are involved in the adaptation of mycobacteria to intracellular environments.

### Rifampicin and ciprofloxacin affect the binding activity of the TetR-like regulator

**Figure 3:**
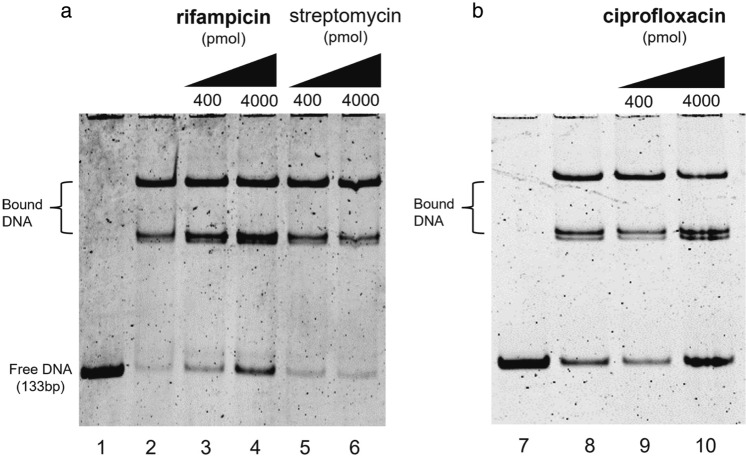
Drug interference in repressor/operator interactions. Binding of the TetR-like MSMEG-3765 to the *MSMEG_3762/63/65* operator region is perturbed in the presence of rifampicin (**a**) or ciprofloxacin (**b**), but not streptomycin (**a**). Lanes 1 and 7: 133 bp DNA fragment containing the 36 bp palindromic motif upstream of *MSMEG_3762*. Lanes 2 and 8: DNA and purified MSMEG-3765, showing low abundance of free operator DNA in the presence of the regulator. In the other lanes, increasing concentrations (400 or 4000 pmol) of rifampicin (lanes 3 and 4) or ciprofloxacin (lanes 9 and 10) show an antimicrobial drug dose-dependent increase in free operator DNA, which does not occur after addition of streptomycin (lanes 5 and 6). The experiment was repeated 3 times.

To further investigate the role of rifampicin and ciprofloxacin in the de-repression of the *MSMEG_3762/63/65* operon, EMSA analyses were performed. The assay measured the binding of the MSMEG-3765 TetR recombinant protein to the cognate *M. smegmatis* 133 bp DNA fragment containing the operator site (36 bp palindromic motif)^20^ in the presence and absence of these antimicrobial drugs. The presence of increasing concentrations of rifampicin or ciprofloxacin (Fig. 3a and b, respectively) in the DNA/protein binding reaction mix resulted in an increase of the free unbound DNA fragment in respect to the control mix without drug. Streptomycin, which the null mutant *ΔMSMEG_3763* was not hypersensitive to (unlike rifampicin and ciprofloxacin, Supplementary Table S2), was used as a negative control. As expected, the MSMEG-3765 binding to the TetR operator sequence was not affected by the presence of streptomycin (Fig. 3a, lanes 5 and 6). These data indicate that rifampicin or ciprofloxacin are able to directly interfear with the binding of the MSMEG-3765 TetR protein to its operator in the *MSMEG_3762/63/65* operon, suggesting their role as specific ligands for this efflux pump repressor.

### The dimer of MSMEG-3765 exhibits the canonical structural architecture of TetR family transcriptional regulators

To understand the biological activities of the MSMEG-3765 repressor, description of its structural and dynamic features is essential, because the shape and nature of the protein surface play crucial roles in protein function. Therefore, we computationally predicted its three-dimensional structure using I-TASSER software (see “Materials and methods”, and Supplementary methods). The Ramachandran plot in Supplementary Fig. S1 reported over 97% residues in most favored and additional allowed regions demonstrating the good quality of the predicted 3D structure.

**Figure 4:**
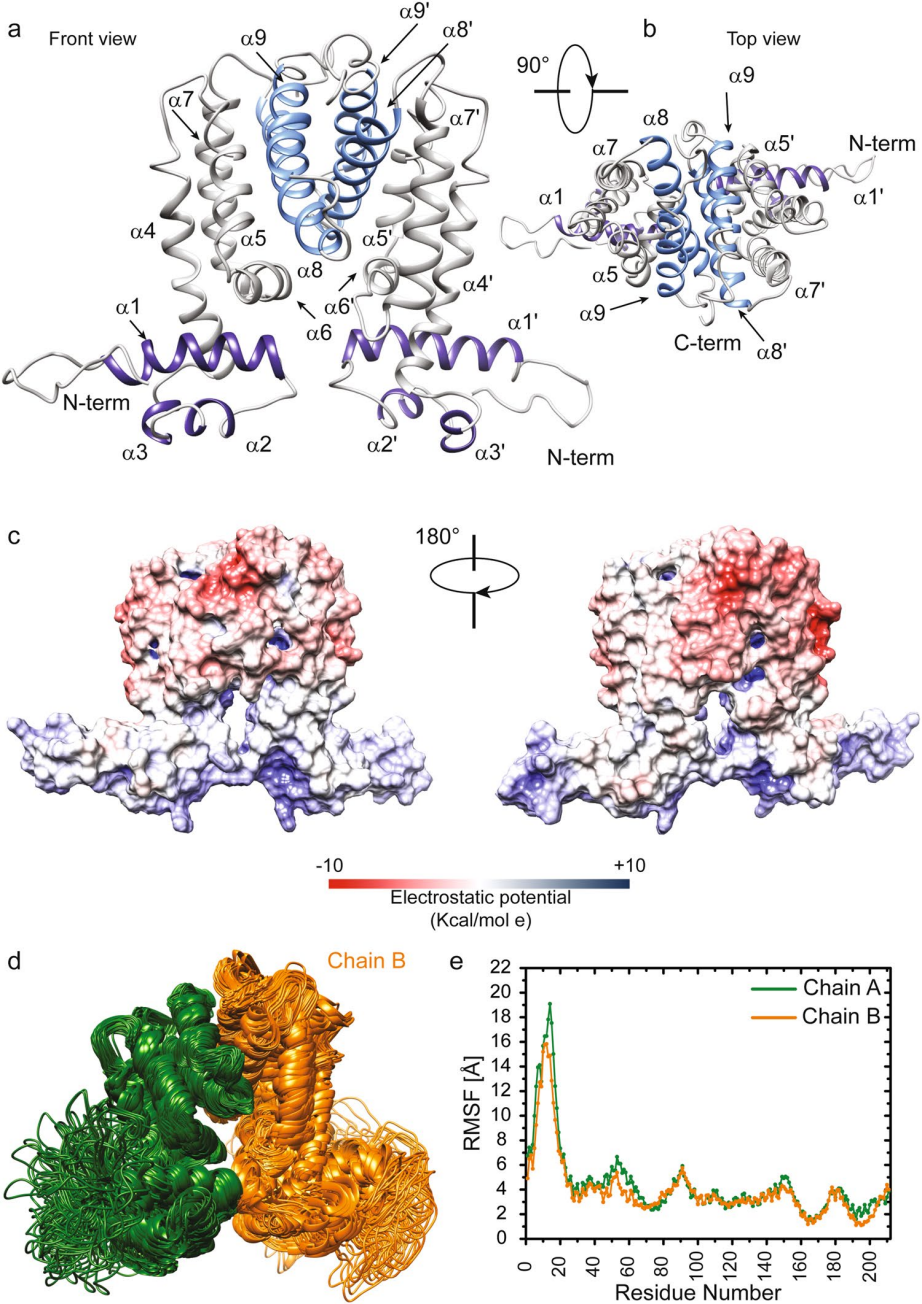
Structure and dynamics of the MSMEG-3765 homodimer. (**a**,**b**) The representative 3D structural model of the MSMEG-3765 transcriptional regulator obtained using the **MSMEG-3765** primary sequence (https://zhanggroup.org/TACOS/) see “Materials and methods”). The helices 1 to 3 of NTD are depicted in dark violet, whereas helices 8 and 9 from each monomer forming a four-helical bundle that makes up the dimer interface are in light blue. (**c**) Electrostatic surface potential map of MSMEG-3765. The MSMEG dimer surface is depicted from electropositive (blue; 10 kcal/mol) to electronegative (red; − 10 kcal/mol). (**d**) Conformational ensemble of the MSMEG-3765 homodimer obtained using NMSim methodology. The two monomers are colored dark green (chain A) and orange (chain B). (**e**) Cα RMSFs (Root Mean Square Fluctuations) plotted versus the primary sequence for MSMEG-3765 chain A and B.

This latter was further validated, on the level of the secondary and quaternary structure organization, by using experimental Circular Dichroism (CD) data (Fig. S2) that were not used in the computational modelling. We compared the α-helix content estimated through the CD spectrum (Fig. S2A,B) with that obtained, as reported in the materials and methods, from the MSMEG-3765 homodimer model. As illustrated in Fig. S2B, the CD data indicate that the α-helix amount of the MSMEG-3765 is 60% which is in a good agreement with the secondary structure content calculated from the 3D structure (63%). Successively, we investigated the accuracy of the homodimer model in the description of the structural peculiarities of MSMEG-3765 by comparing the melting temperature (TM) estimated by CD thermal unfolding (Fig. S2C) and the acquired CD spectrum (S2 A,D,E) and with back-calculated data obtained from the MSMEG-3765 structures predicted for the monomeric and dimeric forms, respectivily. As reflected by the TM and RMSD values (Fig. S2C–E), the dimeric MSMEG-3765 model provides a better description of the experimental CD data than the monomeric form demostrating, together with the EMSA results, that the trascriptional regulator functions as dimer. The overall structural model of MSMEG-3765 is an Ω shaped-dimer (Fig. 4a) typical of a TetR family transcription regulator^27,28^. Each polypeptide chain (residues 2–211) of the MSMEG-3765 homodimer is folded into nine α-helices (α_1_-α_9_ for chain A; α_1_´-α_9_´ for chain B) with connecting turns and loops. The tertiary structure of the MSMEG-3765 monomer is mainly stabilized by hydrophobic helix-to-helix interactions. As for other members of the TetR family, the global structure of the MSMEG-3765 homodimer can be divided into two DNA-binding domains at the N-terminal tail (NTDs) of each monomer, and a regulatory core at the C-terminal domain (CTD) involved in dimerization and ligand binding. Each NTD is composed of helices α_1_ to α_3_ (α_1_ = Ser^21^—Arg^37^; α_2_ = Ile^45^—Ala^50^; α_3_ = Pro^56^—His^60^) (Fig. 4a and b) within which α_2_ and α_3_ constitute the classical helix-turn-helix (HTH) motif that is stabilized by the first α-helix (α1)**.** Moreover**,** helix 1 is preceded by a positively charged region that, as observed for other TetR family members, may have a crucial role in the DNA recognition mechanism^27^. The regulatory domain is formed by helices α_5_ to α_9_ (residues α_5_ = Pro^100^—Ser^109^_,_ α_6_ = Lys^113^—Ile^122^, α_7_ = Val^128^—Asp^148^, α_8_ = Gly^154^—Glu^176^, α_9_ = Val^185^—Gln^197^) and their symmetric helices α_5_´ to α_9_´ and it is connected to the NTDs through helices α_4_ and α_4_´. Helices α_5_ to α_7_ and their symmetric counterparts α_5_´ to α_7_´ form a central triangle subdomain; whereas α_8_ and α_9_ and their symmetric helices α_8_´ and α_9_´ make up a four-helix bundle that constitute the dimerization interface (Fig. 4a and b). Analysis of the electrostatic surface potential indicated that the NTDs are rich of positively charged residues; on the contrary, the core of the regulatory domain is negatively charged with several hydrophobic pockets (Fig. 4c). We then analyzed the conservation of the MSMEG-3765 dimer structure with the Consurf server using as a query the chain A of the predicted homodimer. As expected, the residues located in the HTH motifs of the NTDs were highly conserved (Supplementary Figs. S3, S4). In contrast, the residues situated at the outer surface surrounding the central triangle of the regulatory domain presented low conservation scores. Interestingly, the most conserved residues of the regulatory core domain belong mainly to helices forming the dimerization interface (α_8_, α_9,_ α_8_´ and α_9_´), helices α_5_ to α_7_ and the dyad-related α_5_´ to α_7_´ that, as for other TetR regulators^27^, may play important roles in ligand recognition and modulation of MSMEG-3765 homodimer binding.

### The MSMEG-3765 N-terminal DNA domains are more dynamic than the C-terminal regulatory core domain

In order to understand the relationship between structure, dynamics and function of the MSMEG-3765 homodimer, we investigated the dynamic personality of the dimer using Molecular Dynamics (MD) simulation methods (Fig. 4d and e). These techniques detail the motions and conformational changes of proteins and nucleic acids^29^. We first generated a conformational ensemble describing protein intrinsic motions for the MSMEG-3765 dimer (Fig. 4d); we then analyzed the per-residue RMSF (Root Mean Square Fluctuation) values calculated from the simulated MD ensemble (Fig. 4e). The RMSFs indicate that in each MSMEG-3765 monomer the N-terminal DNA-binding domains (NTD) (residues 1–65) and the C-terminal regulatory domain (CTD) (residues 85–211) are characterized by a different conformational mobility, with the NTDs showing a higher degree of flexibility than CTD (RMSF_avg_^NTD^ = 7.17 ± 3.27 Å; RMSF_avg_^CTD^ = 3.25 ± 0.70 Å) (Fig. 4e and Supplementary Table S3). In particular, in both NTDs the region preceding the first α-helix (residues 1–20) presents higher RMSFs than the helices α1, α2 and α3 demonstrating that the N-terminal tail is more flexible than the rest of the domain. Helix α_4_ and its symmetric counterpart α_4_´, showing a per-residue averaged RMSF of 2.95 ± 0.39 Å and 3.06 ± 0.45 Å, respectively, are connected to the CTD by two ten-residue relatively flexible loops. Helices α_5_ to α_7_ and their symmetric counterparts α_5_´ to α_7_´ present higher RMSF values than the helices involved in the dimerization interface, demonstrating that the four-helix bundle is more rigid than the central triangle subdomain.

### MSMEG-3765/DNA recognition is regulated by interactions between the HTH motifs and the major groove

**Figure 5:**
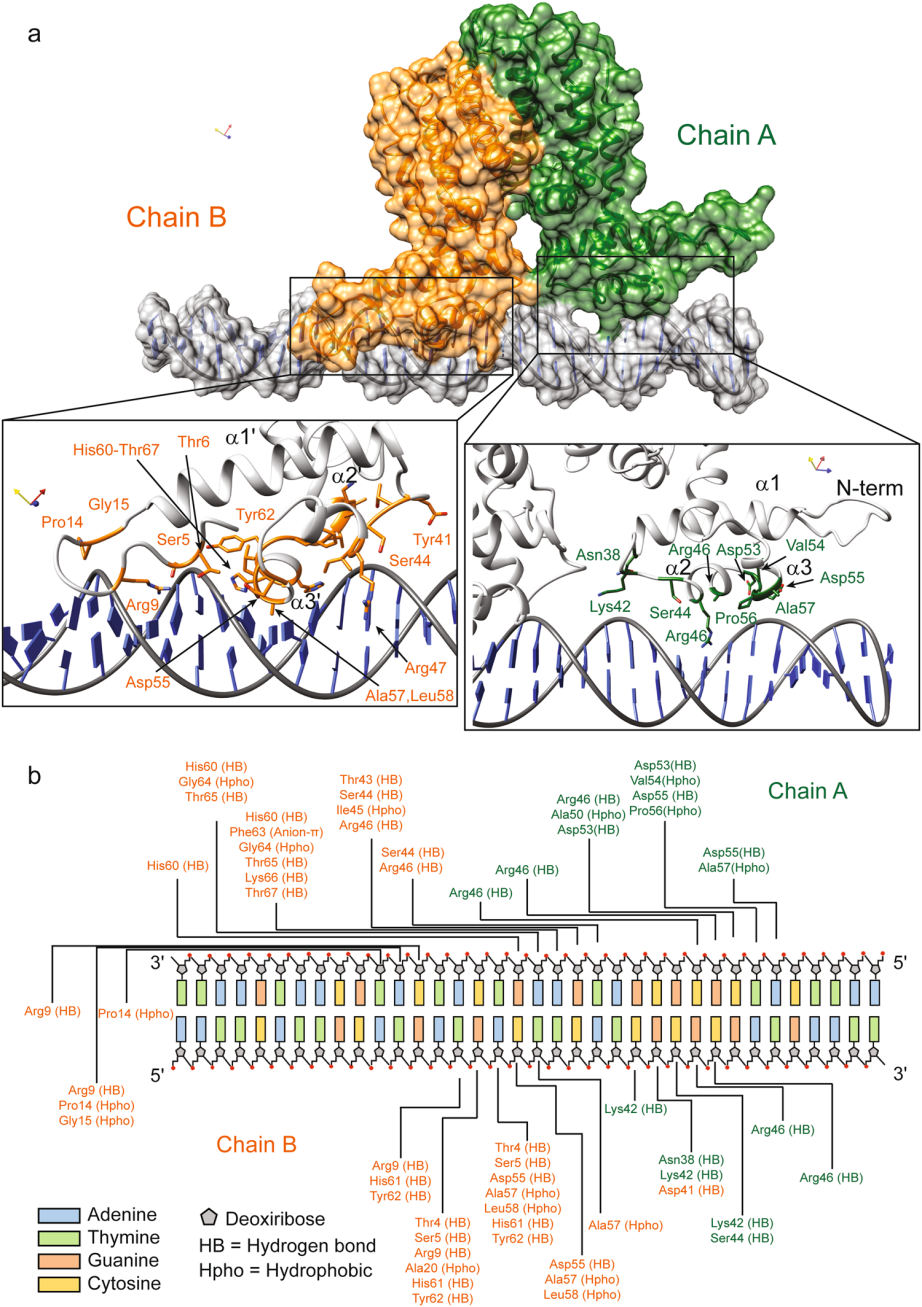
MSMEG-3765/DNA complex interactions. (**a**) Surface representation of the MSMEG-3765 homodimer in complex with DNA as reported by docking studies. Monomer A and B are colored dark green and orange, respectively; the DNA is depicted in light grey. Close-up views of side chain-DNA interactions for the NTD residues involved in the complex formation are detailed. (**b**) A schematic drawing of MSMEG-3765/DNA interactions. Only direct contacts between the protein and the nucleic acid are shown. Hydrogen bond (HB) and hydrophobic interactions (Hpho) are reported. Hydrophobic interactions were determined using a threshold distance of 4.5 Å.

To explore the molecular determinants driving the DNA recognition mechanism of MSMEG-3765 homodimer, we performed a series of molecular docking studies. At the N-terminal region of each MSMEG-3765 monomer the DNA-binding domain (NTD) is composed mainly of the first three helices. According to this observation, the structural model of the MSMEG-3765 dimer/DNA complex shows that the core of each NTD is composed of helices α1- α3, with helix α3 responsible for the majority of DNA contacts (Fig. 5a and b). Helix α3 and the symmetric counterpart α3´ recognize mainly major grooves. Interestingly, the MSMEG-3765 dimer-DNA binding mechanism is mainly driven by phosphate backbone contacts with a significant contribution of base specific interactions in the complex stabilization. Indeed, because the operator sequence is partially palindromic the two NTDs (chain A = 1–65; chain B = 212–276) present a slightly different DNA binding interface producing a slight inclination (~ 15°) of the dimer with respect to the DNA long axis.

In particular, in monomer A, Asn^38^, Lys^42^ and Ser^44^ contact the phosphate backbone of C^24^, G^25^ and G^26^; in helix α2 Arg^46^ forms hydrogen bonds with the phosphate backbone of G^27^, C^28^, C^8^´and G^9^´ (symbol (´) indicates the anti-parallel operator strand) whereas Ala^50^ participates in DNA-binding by making hydrophobic contacts with C^8^´; Ala^57^, located within the recognition helix α3, is involved in hydrophobic base specific interactions fundamental for the DNA binding mechanism. In addition, Asp^53^, Val^54^ and Asp^55^ residues, located inside the turn of the HTH motif of the NTD monomer A, contribute to stabilization of the MSMEG-3765 dimer/DNA complex. For monomer B, in helix α3 Ala^57´^ and Leu^58´^ are involved in hydrophobic interactions with A^17^, C^18^ and T^19^ whereas His^60´^ is hydrogen-bonded with the phosphate backbone of A^17^´, A^18^´ and G^19^’; Ile^45´^ and Arg^46´^ of helix α3 form hydrophobic and hydrogen bond interactions with G^16^´of the anti-parallel DNA strand. Moreover, other interactions between the monomer B and the DNA are hydrogen bonds involving several residues located in the turn of the HTH motif and amino acids situated at the edge of helix α4. Notably, different to chain A, the DNA-binding of monomer B is further stabilized by a positively charged region preceding the first helix of the NTD. In particular, the side chain of Arg^9^ is deeply buried in the minor groove, forming hydrogen bonds with the phosphate backbone of T^15^, and G^16^, C^24^´ and A^25^´ (Fig. 5b).

### Rifampicin and ciprofloxacin bind the MSMEG-3765 repressor at different binding sites

**Figure 6:**
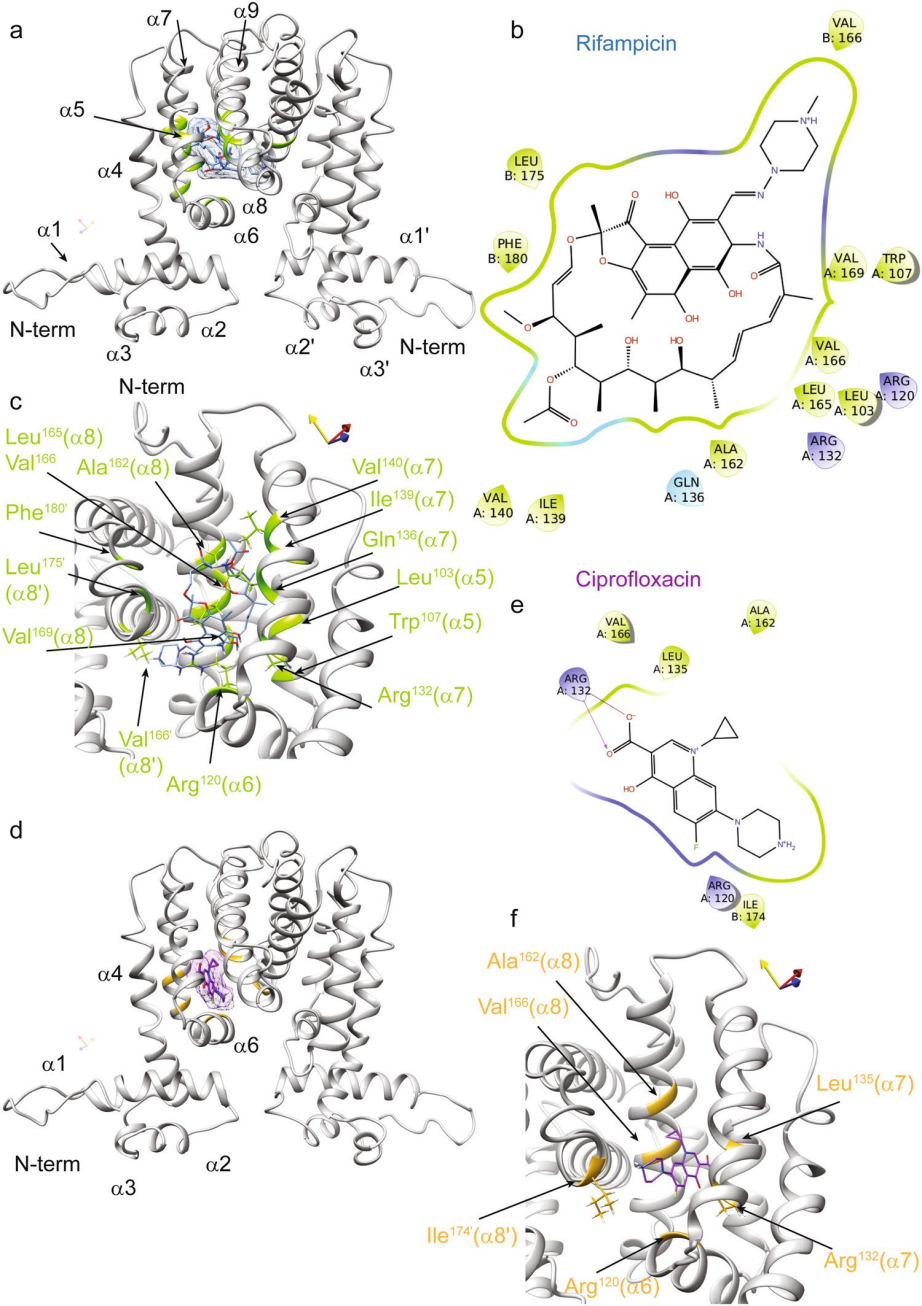
Interactions of the MSMEG-3765 homodimer with rifampicin and ciprofloxacin as revealed by molecular docking. (**a**) Ribbon drawing representation of the MSMEG-3765 dimer/rifampicin complex, where the ligand surface is highlighted as light blue and the regions of the transcription regulator are depicted in light green. (**b**) A 2D interaction map of rifampicin in complex with the MSMEG-3765 dimer identifying key interaction sites. (**c**) The rifampicin binding pocket in chain A of the MSMEG-3765 dimer. The side chains of the MSMEG-3765 residues involved in hydrogen bonds are illustrated as light green, whereas the ligand is colored light blue. The side chains of the residues making hydrophobic interactions are not shown. (**d**) The ciprofloxacin-binding site in chain A of MSMEG-3765. The ligand is reported in magenta, whereas the residues involved in the interactions are colored gold. The surface of the ligand is also shown. (**e**) A 2D interaction map of ciprofloxacin in complex with the MSMEG-3765 dimer. (**f**) Detailed view of the MSMEG-3765/ciprofloxacin complex. The ligand and side chains of the residues involved in the formation of the complex are depicted in magenta and gold, respectively.

To uncover how rifampicin and ciprofloxacin might interact with the TetR-like regulator to abrogate DNA binding, we integrated these antibiotics into our molecular docking models of MSMEG-3765. The 3D structural model of MSMEG-3765 homodimer/rifampicin indicates that the ligand-binding surface of the homodimer is composed of residues from both monomers located within the C-terminal regulatory domain (CTD). Rifampicin binds into a hydrophobic groove of the protein with specific contacts to helices of the central triangle subdomain (α5–α7) and the helix α8 (Fig. 6a). In detail, the binding of rifampicin is principally driven by an extensive network of hydrophobic interactions involving: Leu^103^ and Trp^107^ located in the middle of the α5; Gln^136^, Ile^139^ and Val^140^ encompassing the helix α7; Ala^162^, Leu^165^, Val^166^ and Val^169^ situated within the helix α8; Leu^175^ and Phe^180^ located in the helix α8´ of the other monomer (chain B). Additionally, the interaction of rifampicin with MSMEG-3765 is further stabilized by a series of hydrogen bonds between the ligand and different MSMEG-3765 residues: Arg^120^ at the C-terminal edge of the helix α6; Arg^132^ and Gln^135^ located inside the helix α7; and Ala^162^, Val^166^ of the helix α8 (Fig. 6b and c). In the case of ciprofloxacin, the structure of the MSMEG-3765 homodimer/ciprofloxacin complex shows that the ligand is accommodated into a shallow hydrophobic pocket on the protein surface formed mainly by Leu^135^ of the helix α7 and by two residues of the helix α8 (Ala^162^ and Val^166^).

Moreover, the guanidinium of Arg^132^ (α7) hydrogen binds with both carboxyl oxygens of ciprofloxacin (Fig. 6d–f). These latter interactions play an important role in the stabilization of the MSMEG-3765 homodimer/ciprofloxacin complex. Notably, comparison of the structural models obtained for rifampicin and ciprofloxacin clearly shows that the two antibiotics bind the MSMEG-3765 CTD at distinct binding sites suggesting that the two drugs likely present different structural mechanisms that mediate operon de-repression. To provide structural insight into the potential molecular mechanisms of efflux pump operon de-repression mediated by rifampicin and ciprofloxacin, we analyzed the 3D models of MSMEG-3765 in complex with either drug using the structural data available for other TetR family members^27^. This conformation analysis indicated that, upon ligand binding to CTD, de-repression of the operon likely occurs through conformational changes that abolish the ability of the homodimer to recognize the operator sequence by increasing the separation between NTDs of the two monomers.

## Discussion

Antimicrobial resistance (AMR) of bacterial isolates from patients is a major worldwide concern^30^. Indeed, an increasing number of pathogens are resistant to multiple antibiotics used in routine therapy. AMR is often associated with treatment failure and increased rate of mortality for many infectious diseases, including tuberculosis^31^. Bacterial mechanisms that counteract the impact of antimicrobial compounds are multiple and may generate different degrees of drug tolerance and drug resistance. Mechanisms leading to drug tolerance/phenotypic drug resistance in *M. tuberculosis* include enhanced activity of efflux pumps that remove drugs or toxic intermediates^15^. Many *M. tuberculosis* MDR clinical isolates show mutations in target genes along with overexpression of specific efflux pumps. Machado and coauthors^18^ demonstrated that inhibition of specific efflux pumps in *M. tuberculosis* MDR strains lead to decreased MICs for rifampicin and ofloxacin, first and second line anti-TB drugs, respectively.

In our previous studies on the *M. smegmatis* MSMEG-3762/63 efflux pump, homologous to *M. tuberculosis* Rv1687/86c, we demonstrated that expression of the pump is regulated by the TetR-like MSMEG-3765 repressor, which is also capable of regulating the homologous *Rv1687/86/85c* operon in *M. tuberculosis*^20^. The *M. smegmatis* (*ΔMSMEG_3763*) efflux pump deletion mutant showed lower MICs for rifampicin and ciprofloxacin in comparison to the isogenic wild type and complemented strain, indicating that the extrusion of these two drugs through the MSMEG-3762/63 pump was likely^21^.

Here, we show that the *ΔMSMEG_3763* null mutant exhibits a larger membrane potential than wild type, suggesting that the pump may be involved in both drug and ion transport, as described for other ABC pumps^24,25,32^. For example, the YbhFSR, an ABC exporter in *E. coli*, is a drug efflux pump and a Na + (Li +)/H + antiporter^24^. Moreover, many efflux pumps in *M. tuberculosis* have been associated with more than one anti-TB drug^12,31^. However, no change in drug MIC was found for the MSMEG-3762/63 pump null mutant for other first and second line anti-TB drugs. The efflux pump operon was expressed twofold higher in *M. smegmatis* after macrophage phagocytosis, suggesting that induction of this pump is triggered in response to intracellular conditions. This hypothesis is supported by previous observations showing upregulation of this operon in *M. smegmatis* challenged with acid-nitrosative stress in vitro^20,26^. Other studies have identified differential regulation of efflux pumps after macrophage and/or drug treatment. For example, the overexpression of the Rv1273c ABC efflux pump increased *M. tuberculosis* survival in macrophages^33^. Moreover, Canezin and coauthors^34^ demonstrated that six ABC efflux pumps (Rv1456c, Rv1457c, Rv1458c, Rv1218c, Rv1217c, Rv1819c) were upregulated *in M. tuberculosis* exposed to rifampicin within macrophages. Therefore, we investigated if the MSMEG-3762/63 efflux pump was induced after exposure of *M. smegmatis* to sub-inhibitory concentrations of rifampicin or ciprofloxacin. The results showed that these anti-TB drugs acted as inducers of the efflux pump operon, and this correlated with a decrease in DNA binding activity of the MSMEG-3765 transcriptional repressor in the presence of increasing concentrations of rifampicin or ciprofloxacin, but not streptomycin. These results were acquired using *M. smegmatis *in vitro; future work will reveal whether this mechanism is conserved in *M. tuberculosis*, and whether de-repression of this efflux system by antimicrobial drugs is functionally significant in animal models of TB disease.

To further investigate the mechanism regulating pump activity, we explored the structural and dynamic features of the transcriptional repressor and the molecular forces driving the recognition of DNA and ligands by MSMEG-3765. The 3D structural model of the MSMEG-3765 homodimers revealed that the repressor presents as a Ω shaped organization, characteristic for TetR family members^27,28^, in which each monomer is folded into nine α-helices (α_1_- α_9_ and α_1_´- α_9_´, respectively). Specifically, the MSMEG-3765 dimer consists of two relatively flexible N-terminal DNA binding domains (NTDs) (α_1_—α_3_ and α_1_´- α_3_´, respectively) that are connected, through the α_4_ and α_4_´ helices, to the compact ligand-binding/dimerization C-terminal domain (CTD), which is formed by helices α_5_- α_9_ and α_5_´-α_9_´. Based on the docking model of the MSMEG-3765 dimer/DNA complex, the recognition mechanism is principally driven by the two classical HTH motifs of the NTDs (α_2,_ α_3_ and α_2_´, α_3_´, respectively) with the recognition helices α_3_ and α_3_´ that fit into the DNA major groove. Interestingly, the binding of MSMEG-3765 to the partially palindromic DNA operator sequence is further stabilized by a positively charged region, preceding helix 1 of chain B, that makes contacts with the DNA minor groove; as a result, the homodimer is tilted around 15° with respect to the DNA long axis. In this scenario, we show that the ability of MSMEG-3765 repressor to recognize the efflux pump operon is abolished in the presence of rifampicin or ciprofloxacin. Docking structural data demonstrate that the two antibiotics bind the MSMEG-3765 repressor at different drug-binding sites within a single binding cavity, suggesting that the two drugs mediate de-repression through similar but not identical mechanisms. Rifampicin, in particular, is deeply embedded inside a binding pocket formed by the three helices of the central triangle subdomain, whereas ciprofloxacin binds into a shallow groove at the surface of the repressor. Comparison of the structural data obtained for drug binding with those reported for other TetR family members^27^, suggests that the operon activation caused by rifampicin may occur through a de-repression mechanism very similar to that observed for SimR in which the distance between the two DNA-binding domains increases due to a rigid-body motion of the two monomers relative to each other. Instead for ciprofloxacin the conformational analysis suggests that the de-repression mechanism may involve larger local structural displacements than those observed for rifampicin in several regions of the regulatory C-terminal domain. Extensive studies will be required to fully confirm our in vitro and in silico data; however this modelled scenario so far best explains the molecular and microbiological laboratory observations.

Efflux pumps are widely shown to be important mechanisms responsible for the development of multi-drug resistance in many pathogens. Advances have provided new hope for the design and clinical application of efflux pump inhibitors (EPI) in combination with new antimicrobial drugs to enhance their efficacy^19,35^. An example of a dual-action EPI/antibiotic for *M. tuberculosis* is compound 1599, a derivative of the natural product spectinomycin, which showed more than a 100-fold increased activity against *M. tuberculosis* H37Rv due to reduced susceptibility to efflux by the Rv1258c pump^19^. These observations emphasize the need for further research on this topic and a more thorough understanding of the physiology and structure of efflux pumps to develop complementary and more effective strategies to combat drug-resistant mycobacteria.

## Materials and methods

### Bacterial strains and culture conditions

*Escherichia coli* BL21 (DE3) was used as host for the expression of the TetR-like protein MSMEG-3765. *M. smegmatis* mc^2^155 (wt), *M. smegmatis* mc^2^155 (*ΔMSMEG_3763*) and *M. smegmatis* mc^2^155 (*ΔMSMEG_3763* pBD04) (21) were used throughout this work. *E. coli* was grown in Luria–Bertani (LB) broth, while the *M. smegmatis* strains were cultured in Middlebrook 7H9 broth containing 10% albumin-dextrose-catalase supplement and 0.05% Tween 80. All strains were grown at 37 °C with shaking at 200 rpm. Ampicillin (100 μg mL^−1^) and kanamycin (50 μg mL^−1^ for *E. coli* and 25 μg ml^−1^ for *M. smegmatis*) were used for selection as appropriate. For MICs determination, rifampicin stock solution (10 mg mL^−1^) was prepared in methanol; streptomycin, ethambutol, and isoniazid stock solutions (10 mg mL^−1^) were prepared in filter-sterilized water. Ciprofloxacin, moxifloxacin, levofloxacin and gatifloxacin stock solutions (10 mg mL^−1^) were prepared in 1 M NaOH.

### Measurement of membrane potential

Variations in membrane potential (Δψ) were estimated using a flow cytometry assay based on the *Bac*Light_TM_ Bacterial Membrane Potential Kit (Life Technologies). Cultures of *M. smegmatis* mc^2^155 (wt), *M. smegmatis* mc^2^ 155 (*ΔMSMEG_3763*) and *M. smegmatis* mc^2^155 (*ΔMSMEG_3763* pBD04) were grown to mid-log phase (OD_600_ 0.6), passed through a 25-gauge syringe to disrupt the clumps and diluted in filtered phosphate-buffered saline (PBS) to ~ 1 × 10^6^ cells/mL. To 1 mL of each suspension were added 15 μM of the fluorescent membrane potential indicator DiOC_2_ (3,3’- diethyloxocarbocyanine iodide) and 1 mM of EDTA to facilitate the dye uptake. The samples were incubated at room temperature for 30 min and data were recorded using an Accuri C6 BD flow cytometer, with emission filters suitable for detecting red (488/610 nm) and green fluorescence (488/530 nm). As a positive control for membrane depolarization, cultures were treated with 5 μM protonophore carbonyl-cyanide 3- chlorophenylhydrazone (CCCP). Membrane potential was calculated as a ratio of red/green fluorescence^36,37^.

### RNA extraction and reverse transcription reactions

RNA for RT-qPCR analyses were extracted from *M. smegmatis* mc^2^ 155 grown to log-phase (OD_600_ 0.4) and exposed to sub-inhibitory (sub-MIC) concentrations of rifampicin (1 µg mL^−1^) and ciprofloxacin (0.083 µg mL^−1^), corresponding to 1/3rd in vitro MIC. A drug-free bacterial culture was used as untreated control and all cultures were incubated at 37 °C for a further 1 h. Bacteria were harvested by centrifugation (3273 rcf, 10 min, 4 °C), resuspended in 4 mL RLT buffer (Qiagen), and vortexed using glass beads. Total RNA was extracted using the RNeasy columns (Qiagen) according to the manufacturer's instructions and subsequently treated with RNase-free DNase I (New England BioLabs) for 10 min at 37 °C, followed by phenol/chloroform purification. The quality and quantity of RNA were evaluated using the NanoDrop spectrophotometer (Nanodrop, Thermo Scientific) and gel electrophoresis. Reverse transcription was performed for 15 min at 42 °C in a total volume of 40 μl containing 2 μg RNA (QuantiTect Reverse Transcription kit, Qiagen). Samples without the reverse transcription step were used as negative controls.

### Transcriptional analyses by RT-qPCR

RT-qPCR, targeting the *MSMEG_3762* gene, was performed to evaluate the expression of the *MSMEG_3762/63/65* operon in *M. smegmatis* mc^2^ 155 under standard conditions and after exposure to sub-inhibitory concentrations of rifampicin and ciprofloxacin (oligonucleotides detailed in Supplementary Table S1). Analysis by Real-time PCR was performed using SYBR green technology with a StepOnePlus RT-qPCR system (Applied Biosystems). The *sigA* gene was used as an internal standard for expression analysis. PCR conditions of denaturation at 95 °C for 10 min; 40 cycles of 15 s amplification at 95 °C; 1 min at 60 °C followed by 30 s at 72 °C were used. The transcriptional analysis was performed in triplicate from independent biological replicates. Standard curves for both internal control and target genes were obtained by amplifying serial dilutions (ratio 1:10) of the samples. Relative expression levels were normalized using *sigA* and calculated using the Pfaffl method relative to drug-free bacilli^38^.

### Macrophage culture and infection

Human monocyte THP-1 cells were cultured in RPMI 1640 medium supplemented with 10% v/v heat inactivated sterile-filtered bovine serum (PAN-Biotech) and 1% L-glutamine (2 mM final concentration) at 37 °C in a 5% CO_2_ incubator to a density of 1 × 10^6^ cells/mL. Monocytes (1,5 × 10^7^ total cells) were differentiated into macrophages by treatment with phorbol-12-myristate-13-acetate 200 nM for 24 h at 37 °C and 5% CO_2_. After washing twice with phosphate-buffered saline (PBS), cells were rested for 48 h in RPMI before infection. *M. smegmatis* log phase bacilli were harvested, washed in PBS, re-suspended in an equal volume of RPMI, and syringed 5 times to generate a homogeneous cell suspension, before infecting macrophages at a multiplicity of infection of 10:1 (10 bacilli:1 macrophage) for 2 h in a 5% CO_2_ humidified incubator.

### RNA extraction from phagocytosed Mycobacteria and RT-qPCR

RNA was extracted from phagocytosed mycobacteria as described by Tailleux and coauthors^39^. After washing off extracellular mycobacteria with PBS, the infected macrophages were lysed using 4 M GTC solution (250 g guanidine thiocyanate, 2.5 g N-lauroyl-sarcosine, 12.5 mL 1 M sodium citrate pH 7.0, 5 mL Tween 80, 3.4 mL β–mercaptoethanol in 500 mL ultrapure water). The bacterial pellet was recovered by centrifugation at 3000 *rcf* for 20 min, resuspended in TRIzol (Thermo Fisher Scientific), and disrupted using a ribolyser with 0.1 mm silica beads (MP Biomedicals) at speed 6.5 for 45 s. The RNA samples were DNase-treated and purified using RNeasy columns (Qiagen). RNA quality and yield were evaluated using the Nanodrop One (Thermo Scientific, Waltham, MA, USA) and Agilent 2100 Bioanalyzer (Agilent Technologies, Santa Clara, CA, USA). RT-qPCR analyses were performed to evaluate the expression of *MSMEG_3762* in macrophage-phagocytosed bacilli using the oligonucleotides RTMSMEG3762f/RTMSMEG3762r (Supplementary Table S1), with *sigA* as an internal control. The reverse transcriptase and the Real-time PCR were performed using the QuantiNova SYBR green RT-PCR kit (Qiagen) that allows both reactions to take place in a single tube, set up according to the manufacturer's instructions. The transcriptional analysis was performed on three independent biological replicates and fold change calculated using the 2^−ΔΔCt^ method relative to bacilli resuspended in RPMI^40^.

### Electrophoretic mobility shift assay (EMSA)

Electrophoretic Mobility Shift Assay analyses were performed to evaluate the binding activity of the MSMEG-3765 recombinant protein to the upstream region of the *MSMEG_3762/63/65* operon in the presence of putative effectors (rifampicin, ciprofloxacin and strptomycin). The recombinant MSMEG-3765 protein was purified as previously described^20^. The final step of purification was performed using Protino NI-TED-IDA 2000Kit (MACHEREY–NAGEL). A 133 bp fragment containing the 36 bp palindromic motif, previously identified as the binding site of the Tet-R like protein, was amplified from *M. smegmatis* mc^2^ 155 using the primer pair mot3762f1/MS14r (Supplementary Table S1)^20^. The reaction mixture (20 μl) contained 0.2 pmol DNA, 4 pmol purified recombinant MSMEG-3765, and increasing concentrations (0–400–4000 pmol) of rifampicin, ciprofloxacin or streptomycin at 30 °C for 10 min. The binding buffer contained 20 mM HEPES pH 8.0, 75 mM NaCl, 10 mM MgCl_2,_ and 5% glycerol. The mixture was loaded onto native PAGE gels (8% acrylamide:bisacrylamide (30:1)) and electrophoresed at 4 °C for 4 h. The gel was stained with SYBR™ Gold Nucleic Acid Gel Stain (Invitrogen) and the results were analyzed with a Typhoon ™ Trio + gel scanner. Streptomycin was used as a negative control drug exposure.

### CD spectroscopy

MSMEG-3765 sample was prepared in 20 mM Tris HCl and 500 mM NaCl at pH 8. The thermal denaturation was carried out using a JASCO-815 CD spectropolarimeter equipped with Peltier temperature control. CD spectra were measured at 10 °C intervals in the 5–85 °C range. The data were collected using a quartz cuvette with a 1 cm path-length in the 200–260 nm wavelength range with a data pitch of 1 nm. All data were acquired with a bandwidth of 1 nm with a scanning speed of 50 nm min-1 and normalized against the reference spectrum in order to remove the buffer background contribution. The CD data were fitted into a two state-state folding model.

### Three-dimensional structure modelling of the MSMEG-3765 homodimer

The 3D structure of the MSMEG-3765 homodimer was predicted using a series of bioinformatics tools through two-step conformational modeling using the software I-TASSER^41,42^ (for details see SI). The quality of the selected representative structural model of the MSMEG-3765 monomer was assessed by analyzing the Ramachandran plot obtained using PROCHECK^43^. The 3D structure of the MSMEG-3765 homodimer was generated by the TACOS server (https://zhanggroup.org/TACOS/), using the I-TASSER conformer of the monomer as template. The TACOS algorithm predicted five models with a C-score ranging from -0.72 to -2.39 for the MSMEG-3765 homodimer. We selected as reference structure the MSMEG-3765 homodimer structural model with the highest C-score and we used it for all structural calculations. The MSMEG-3765 homodimer was visualized and analyzed using PyMol^44^, Chimera^45^ and Consurf (https://consurf.tau.ac.il/). Estimation of the secondary structure was performed using DSSP^46^.

### Validation of the homodimer MSMEG-3765 structural model

The predicted 3D structural model of the dimer was validated by comparing experimental CD structural information with data back-calculated from the MSMEG-3765 structures in monomeric and dimeric forms. The α-helical content was estimated from the deconvolution of CD data using DichroWeb website (http://dichroweb.cryst.bbk.ac.uk/html/home.shtml). The prediction of melting temperatures and CD spectra from the monomer and dimer MSMEG-3765 structural models was performed using SCooP (babylone.ulb.ac.be/SCooP/) and PDBMD2CD (https://pdbmd2cd.cryst.bbk.ac.uk/) software, respectively.

### MSMEG-3765 homodimer dynamics studies

To explore MSMEG-3765 homodimer dynamics a conformational sampling approach was used to produce an ensemble of conformers. The Normal Mode-based Simulation (NMSim) methodology^47^ has been proved a computationally efficient alternative to the conventional MD simulation for conformational sampling. Therefore, starting from the predicted 3D structural model of the MSMEG-3765 homodimer, we generated an ensemble of 2500 conformers with NMSim using the parameters suitable for sampling large-scale motions (further details in SI). The resulting conformational ensemble was clustered using NMRCLUST^48^, generating a final representative ensemble containing 135 conformers.

### Building the MSMEG-3765 homodimer/DNA complex by molecular docking

The three-dimensional structure of the DNA molecule for the docking studies was built using the software package 3DNA^49^ from 36 bp palindromic motif upstream of *MSMEG_3762* used in the EMSA experiments above described (5´ → 3´ strand AATTCATTGCATGATGACTTCATCGCGCGATGAATT). The software HDOCK and MPRDOCK^50^ were used to dock the MSMEG-3765 homodimer to DNA using default docking parameters without adding any information about the binding site. By using this strategy, we obtained ten models for the MSMEG-3765 homodimer/DNA complex and we selected the conformer with the lowest docking energy scores as the representative structure. The molecular interactions involved in the formation of the MSMEG-3765/DNA complex were identified using the server MAPIYA^51^.

### Molecular docking of MSMEG-3765 homodimer/ligand complexes

The software DockThor^52^ was used to dock rifampicin and ciprofloxacin to the MSMEG-3765 transcriptional regulator. DockThor employs a multiple solution genetic algorithm as search method and the MMFF94S force field as the scoring function in order to rank the obtained docking poses. We used our MSMEG-3765 homodimer structure in the docking protocol. The rifampicin and ciprofloxacin 3D structures were downloaded from the LigandBox database^53^. The docking calculations were performed using the following parameters: Grid size x = 25 Å, y = 25 Å, z = 18 Å; discretization 0.25. Hydrogen bonds and hydrophobic interactions were identified and visualized using the PLIP web server^54^ and the software Maestro (BioLuminate, Schrödinger, LLC, New York, NY, 2021).

### Supplementary Information

Supplementary Information.

## Data Availability

All the data that support the findings of this study are available in the paper and its Supplementary information published online.
