# Supplementary material for: Insight into the on/off switch that regulates expression of the MSMEG-3762/63 efflux pump in Mycobacterium smegmatis
Source: Sci Rep. 2023 Nov 21;13:20332. doi: 10.1038/s41598-023-47695-4 (PMC10663510; doi:10.1038/s41598-023-47695-4)
Supplement: Supplementary file 1 — Supplementary Information. [file 41598_2023_47695_MOESM1_ESM.doc]

**Supplementary Information**

**Insight into the on/off switch that regulates expression of the MSMEG-3762/63 efflux pump in *Mycobacterium smegmatis***

**Nicoletta Campolattano^1^, Gianluca D’Abrosca^1,2^, Luigi Russo^1^, Barbara De Siena^1^, Milena Della Gala^1^, Ida De Chiara^1^, Rosangela Marasco^1^, Aaron Goff^3^, Simon J Waddell^3^, Margherita Sacco^1^ and Lidia Muscariello^1*^**

^1^Dipartimento di Scienze e Tecnologie Ambientali Biologiche e Farmaceutiche, Università degli Studi della Campania Luigi Vanvitelli, Caserta, Italy;

^2^ Present address: Department of Clinical and Experimental Medicine, University of Foggia, Foggia, Italy

^3^Department of Global Health and Infection, Brighton and Sussex Medical School, University of Sussex, Brighton, BN1 9PX, United Kingdom;

* Correspondence: [lidia.muscariello@unicampania.it](mailto:lidia.muscariello@unicampania.it)

**Supplementary Methods**

**Description of the I-TASSER protocol used to build the 3D structural model of the MSMEG-3765 monomer**

The 3D structure of the MSMEG-3765 homodimer was predicted using the software I-TASSER. First, the tertiary structure of the MSMEG-3765 monomer was obtained on the basis of the amino acid sequence using the software I-TASSER (Interactive Threading ASSEmbly Refinment). This computational approach represents a powerful tool for protein structure prediction [37] and structure-based annotation that employs the three conventional methodologies for protein structure modelling: comparative modelling, threading and *ab initio* modelling [38]. In the case of the MSMEG-3765 monomer, the I-TASSER algorithm generated five models with a C-scores ranging from -1.30 to -2.71. According to I-TASSER criteria, the model with the highest C-score (C-score -1.30) was used as the representative structure to build the three-dimensional model of the dimer.

**Description of the parameters used to generate the conformational ensemble of the MSMEG-3765 homodimer by NMSim.**

The NMSim calculation was performed using the following parameters: E-cutoff for H-bonds (kcal/mol)= -1; hydrophobic cutoff (Å)= 0.35; hydrophobic method= 3; RCNMA NM-method= RCNMA; cutoff for C-alpha atoms (Å)= 10; NMSim number of trajectory= 5; number of NMSim cycles= 1; side-chain distorsions= 0.05; number of simulation cycles= 500; output frequency= 1; NM modes range= 1-5; ROG mode= none; step size=0.

**Table S1**. Oligos used in this work.

| **Primer** | **Sequence** | **Target gene** | **Application** | **Reference** |
| --- | --- | --- | --- | --- |
| RTMSMEG3762f | 5’-GATCTGTGGCAGCAGTTCAA-3’ | *MSMEG_3762/63* (F) | RT-qPCR | [21] |
| RTMSMEG3762r | 5’-GGAACTGCATGAGGTGTCCT-3’ | *MSMEG_3762/63* (R) | RT-qPCR | [21] |
| RTMSMEG2758f (*sigA*) | 5’-CCAAGGGCTACAAGTTCTCG-3’ | *MSMEG_2758* (F) | RT-qPCR | [21] |
| RTMSMEG2758r  (*sigA*) | 5’-CTTGTTGATCACCTCGACCA-3’ | *MSMEG_2758* (R) | RT-qPCR | [21] |
| mot3762f1 | 5’- CTGCTGGAGTCCGCCGCCTG-3’ | *MSMEG_3762* upstream region (F) | EMSA | [20] |
| MS14r | 5’CGGGCCCCGTCGCTGACAATTCATCGC-3’ | *MSMEG_3762* upstream region (R) | EMSA | [20] |

**Table S2. MICs determination in presence of first and second-line anti-TB drugs.**

| **First-line drugs**  (Ref.) | **streptomycin**  (This work) | **isoniazid**  (This work) | **ethambutol**  (This work) | **rifampicin**  [21] |
| --- | --- | --- | --- | --- |
| *M. smegmatis* mc^2^155 | 1.2 μg/ml | 2 μg/ml | 0.6 μg/ml | 3 μg/ml |
| *M. smegmatis* mc^2^155 (*ΔMSMEG_3763*) | 1.2 μg/ml | 2 μg/ml | 0.6 μg/ml | 2 μg/ml |
| *M. smegmatis* mc^2^155 (*ΔMSMEG_3763* pBD04) | 1.2 μg/ml | 2 μg/ml | 0.6 μg/ml | 3 μg/ml |
| **Second-line drugs**  (Ref.) | **Levofloxacin**  (This work) | **moxifloxacin**  (This work) | **gatifloxacin**  (This work) | **ciprofloxacin**  [21] |
| *M. smegmatis* mc^2^155 | 0.25 μg/ml | 2 μg/ml | 0.06 μg/ml | 0.25 μg/ml |
| *M. smegmatis* mc^2^155 (*ΔMSMEG_3763*) | 0.25 μg/ml | 2 μg/ml | 0.06 μg/ml | 0.12 μg/ml |
| *M. smegmatis* mc^2^155 (*ΔMSMEG_3763* pBD04) | 0.25 μg/ml | 2 μg/ml | 0.06 μg/ml | 0.25 μg/ml |

**Table S3.** Average RMSF values calculated for the nine helices of the two polypeptide chains characterizing the MSMEG-3765 homodimer structure.

| **Chain B** | **Chain A** |  |
| --- | --- | --- |
| 4.20 ± 0.72 | 4.68 ± 0.69 | **Helix 1** |
| 3.74 ± 0.43 | 4.98 ± 0.43 | **Helix 2** |
| 3.51 ± 0.31 | 5.08 ± 0.45 | **Helix 3** |
| 3.06 ± 0.45 | 2.95 ± 0.39 | **Helix 4** |
| 3.24 ± 0.27 | 3.25 ± 0.30 | **Helix 5** |
| 2.87 ± 0.09 | 2.91 ± 0.25 | **Helix 6** |
| 3.36 ± 0.29 | 3.46 ± 0.43 | **Helix 7** |
| 2.21 ± 0.60 | 2.36 ± 0.63 | **Helix 8** |
| 1.76 ± 0.51 | 2.50 ± 0.43 | **Helix 9** |

**Figure S1:** Ramachandran plot of the MSMEG-3765 monomer. The plot statistics are also reported.

**
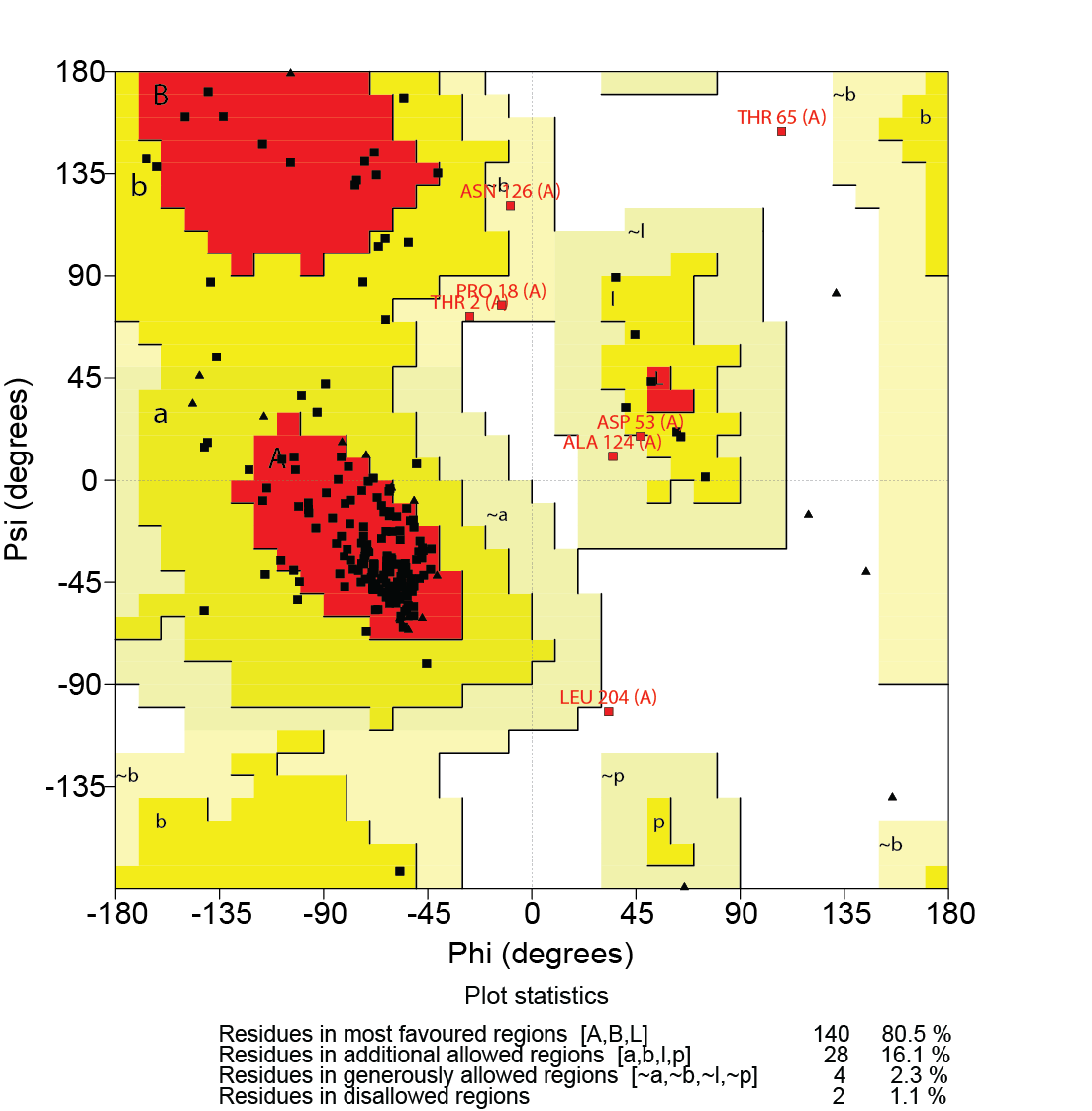
**

**Figure S2:** Circular Dichroism analysis and validation of MSMEG-3765. (A) CD spectrum of MSMEG-3765 acquired at 25 °C. (B) α-helix content estimated from the 3D structural model and from the CD data deconvolution. (C) *Upper* Thermal unfolding experiment of MSMEG-3765 carried out in the range of 5 – 85 °C. Melting curve (blue) of MSMEG-3765 monitored by CD at 222 nm. The data were fitted using a two-state model. *Lower* Comparison of the melting temperature (Tm) obtained by CD with the values estimated using the 3D structural models predicted for MSMEG-3765 in monomeric and dimeric forms. (D, E) Overlay of the CD spectrum measured for MSMEG-3765 (red) with those back-calculated using the 3D structures of monomeric (C) and dimeric (D) MSMEG-3765.

**
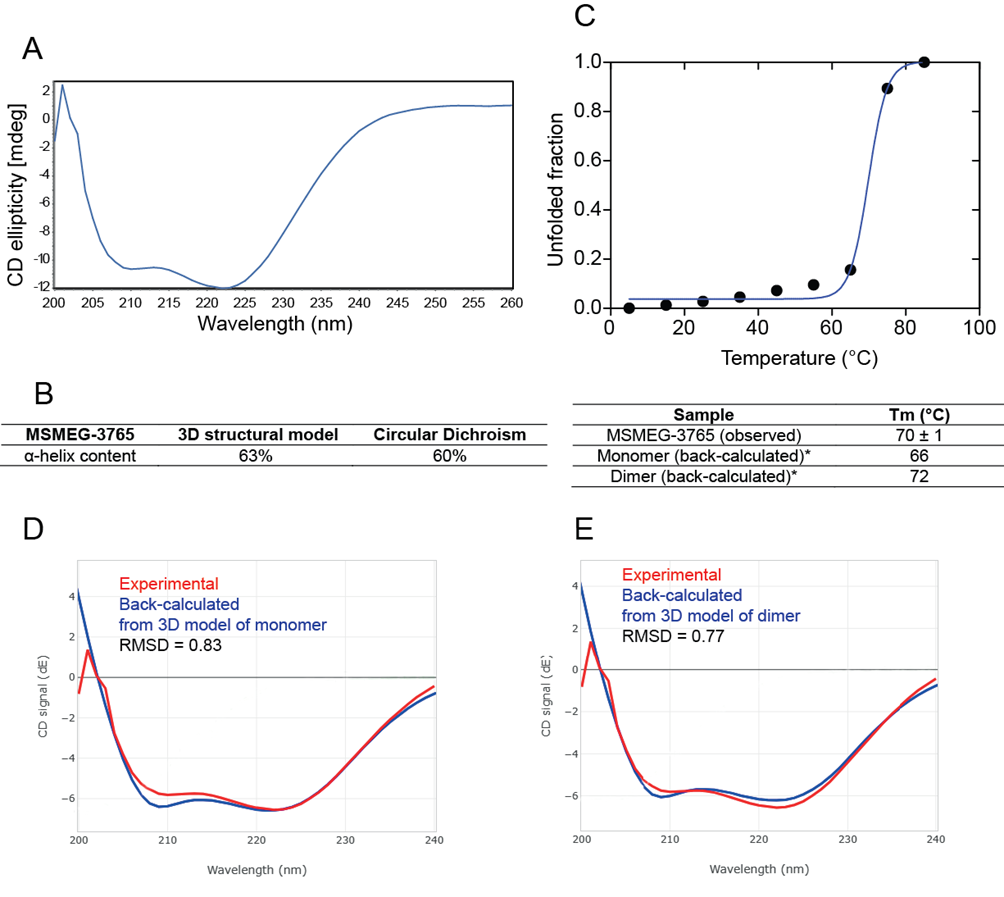
**

**Figure S3:** The conservation of MSMEG-3765 residues based on ConSurf server analysis. Cartoon representation of the chain A of MSMEG-3765 homodimer. The secondary structure elements are depicted according to conservation grades as reported by ConSurf server.

**
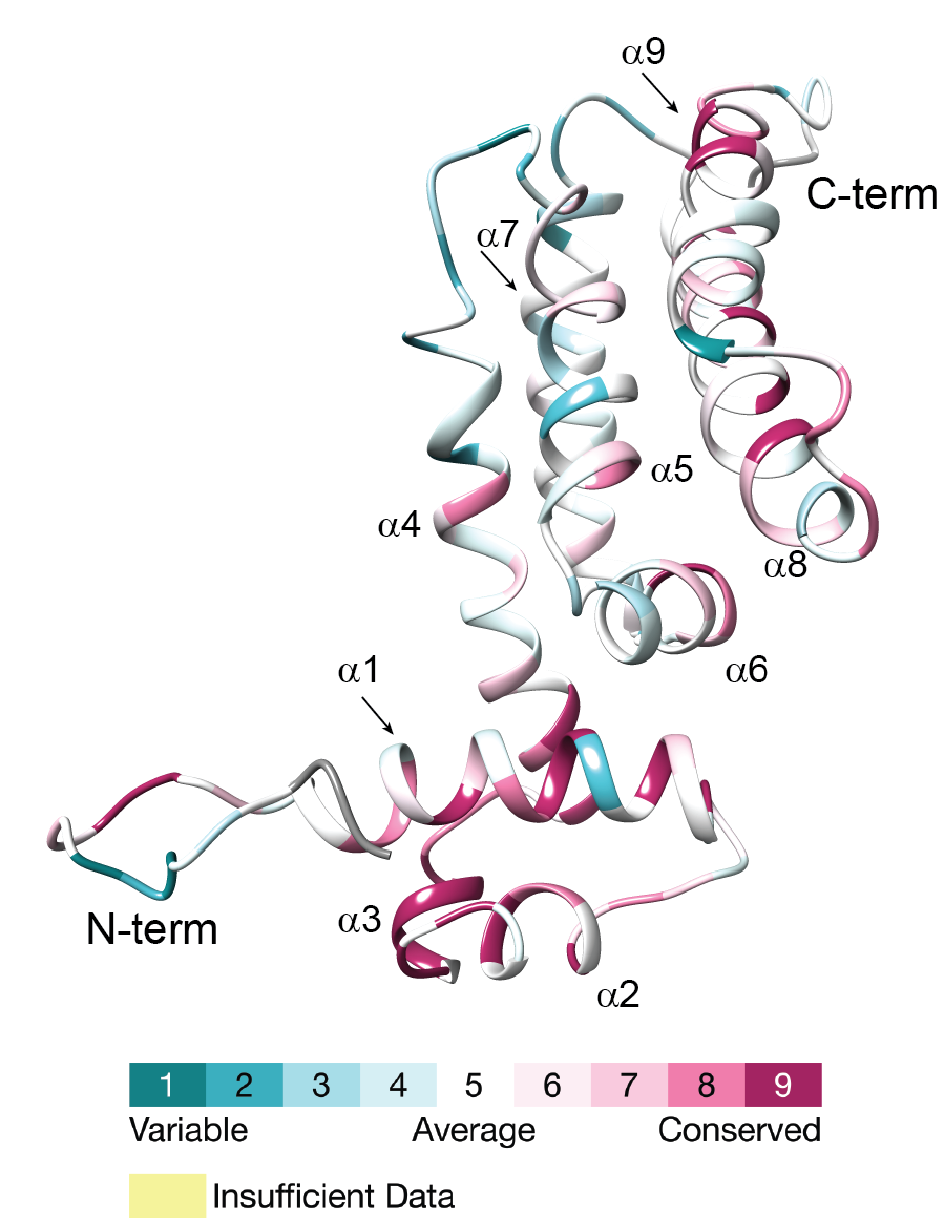
**

**Figure S4:** Statistical data of the ConSurf server analysis.

**
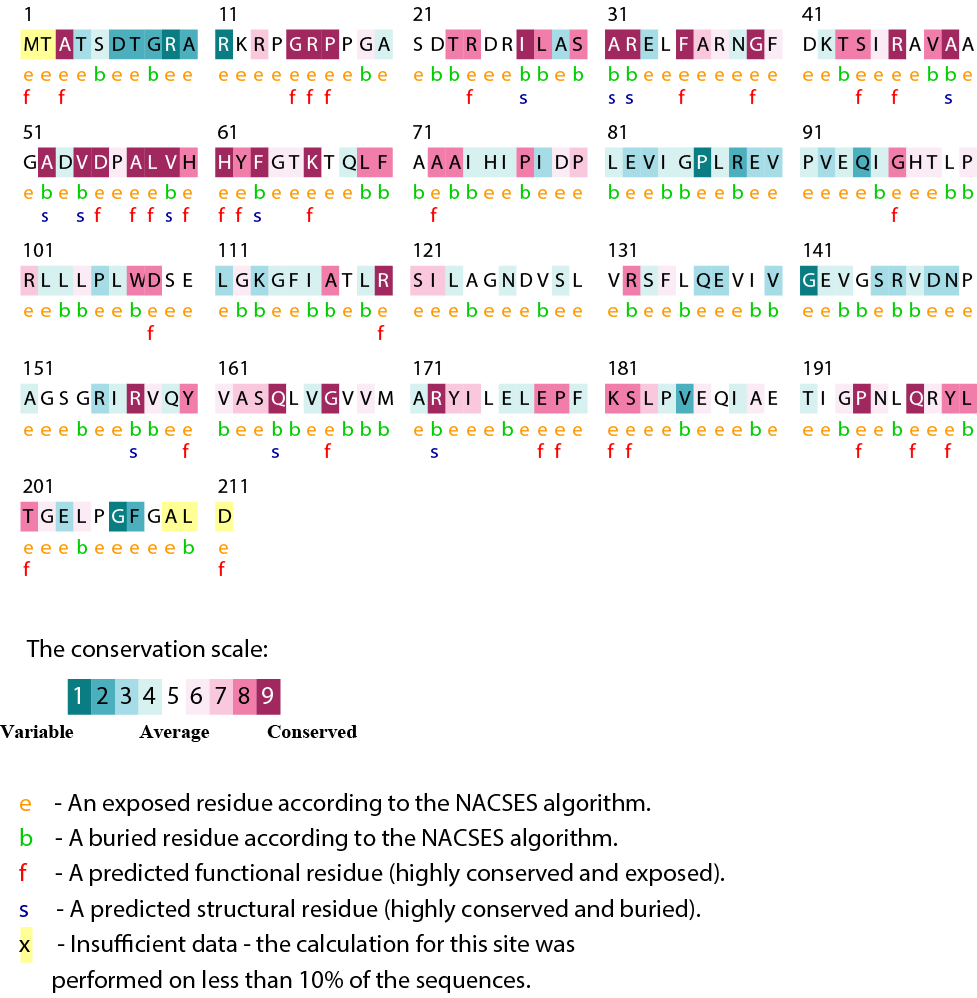
**

**Equipment and settings**

**Original images of EMSA analysis**


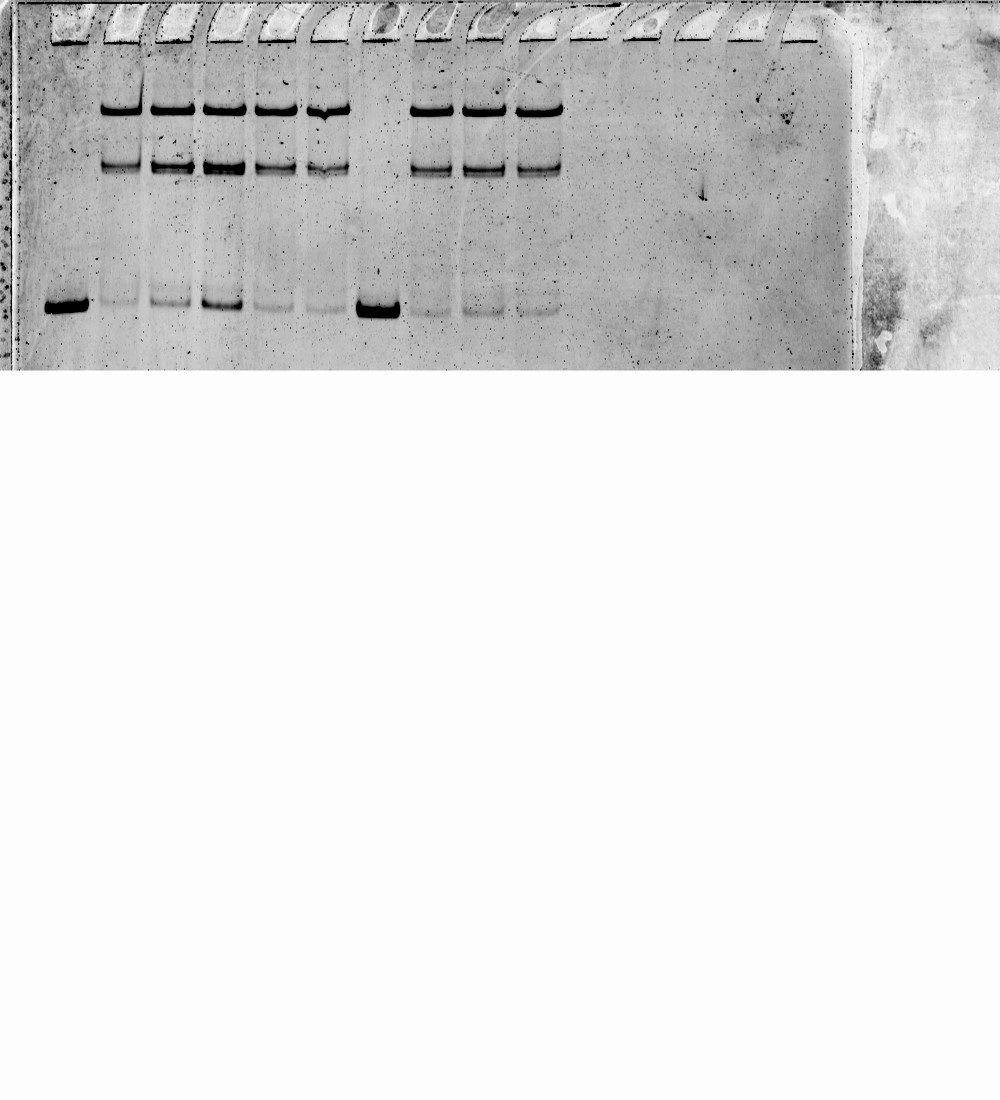


1

2

3

4

5

6

7

8

9

10

133 bp

DNA-MSMEG-3765

complex

**Figure S5. Full length gel-electrophoresis acquisition of the EMSA experiment described in figure 3, panel (a) of the manuscript.**

**Lanes 1 and 7:** 133bp DNA fragment (0.2 pmol) containing the 36 bp palindromic motif upstream of *MSMEG_3762.* **Lanes 2 and 8:** DNA and purified MSMEG-3765 (4.0 pmol), showing low abundance of free operator DNA in the presence of the regulator. In the other lanes, increasing concentrations (400 or 4,000 pmol) of rifampicin (**lanes 3 and 4**), show an antimicrobial drug dose-dependent increase in free operator DNA, which does not occur after addition of streptomycin (**lanes 5 and 6**) or ethambutol (**lanes 9 and 10**). NOTE: in figure 3 of the manuscript lanes 7-10 have been deleted because we chose to report just one negative control (streptomycin) to demonstrate the binding specificity of rifampicin.

The gel was stained with SYBR^TM^ Gold Nucleic Acid Gel Stain (Invitrogen) and the results were analyzed with a Typhoon^TM^ Trio + gel scanner (GE Ealthcare). The image was processed by ImageQuant TL8.1 software (image-gathering settings: filter 520 BP 40, Laser Blue 488nm, Sensitivity: normal, PMT 600V).

The red box indicates the part of the image reported in panel (a) of figure 3 in the manuscript.


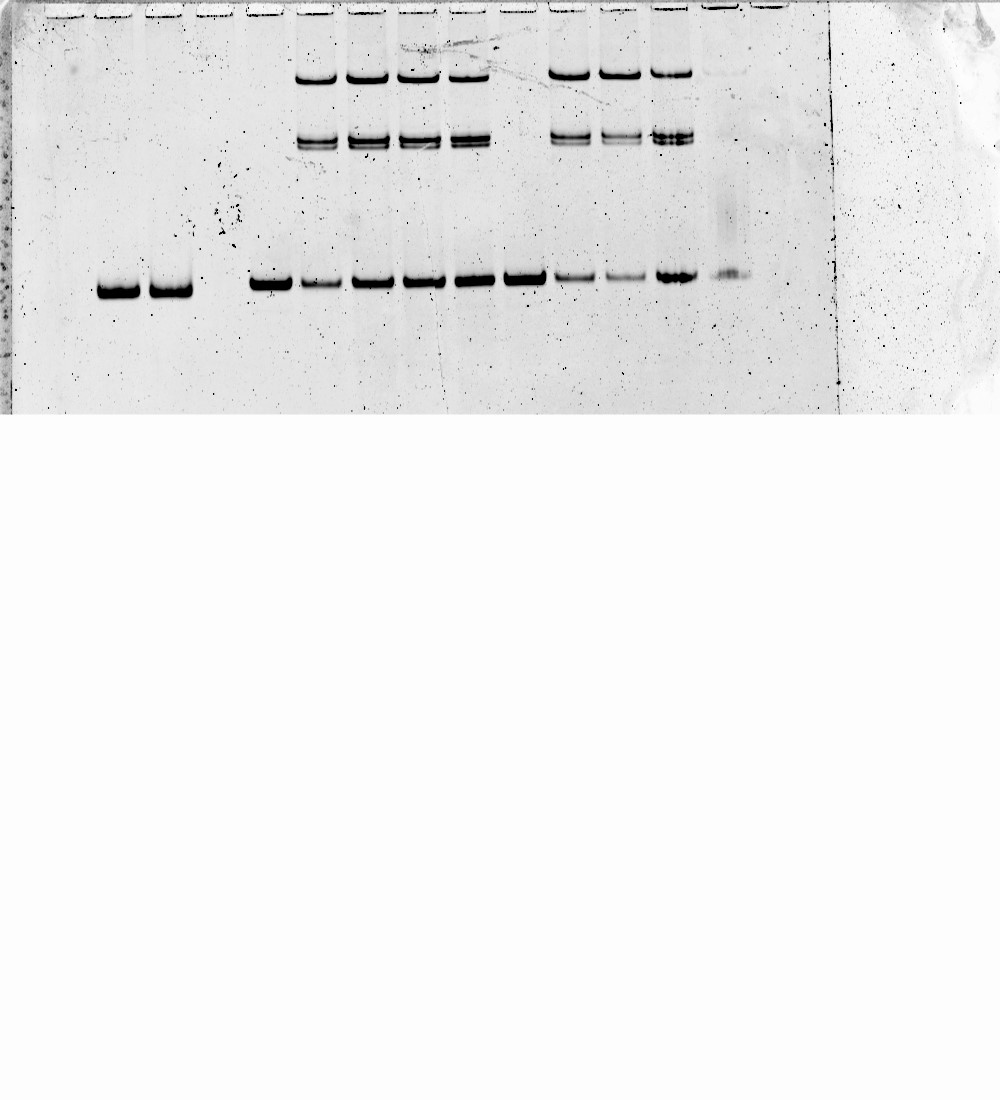


1

2

3

4

5

6

7

8

9

10

11

12

13

127 bp

DNA-MSMEG-3765

complex

133 bp

**Figure S6. Full length gel-electrophoresis acquisition of the EMSA experiment described in figure 3, panel (b) of the manuscript.**

**Lanes 1 and 2:** 127 bp fragment containing the promoter of *MSMEG_3760* used as a negative control for DNA-protein complex formation, (line 1: free DNA; line 2: DNA + 4 pmol of the TetR-like protein, MSEG-3765). **NOTE:** those lanes were excluded from figure 3, reported in the manuscript, given that the specificity of the binding of MSMEG-3765 to the operator sequence was just assessed in a previous work by EMSA analysis including appropriate controls (Perrone et al, 2017, doi:10.3389/fmicb.2017.02039).

**Lane 3:** empty well. **Lanes 4 and 9:** 133bp DNA fragment (0.2 pmol) containing the 36 bp palindromic motif upstream of *MSMEG_3762.*

**Lanes 5 and 10:** 133bp DNA fragment (0.2 pmol) and purified MSMEG-3765 (4.0 pmol), showing low abundance of free operator DNA in the presence of the regulator.

**Lanes 6-8:** DNA and purified MSMEG-3765 protein (4.0 pmol), containing an increasing concentration (100, 200 or 400 pmol, respectively) of rifampicin. **NOTE:** the ability of rifampicin to dissociate the DNA-protein complex (see the increase of the free DNA in lanes 6 to 8) was further investigated by using 400 and 4000 pmol of drug (as shown in figure 3, panel (a) of the manuscript)**.**

**Lanes 11-13:** DNA and purified MSMEG-3765 protein (4.0 pmol), containing an increasing concentration (400, 4000 or 20000 pmol, respectively) of ciprofloxacin. Lane 13 was excluded by the figure 3 reported in the manuscript as it showed an unclear result, probably due to a technical problem within the gel or the electrophoretic run.

The gel was stained with SYBR^TM^ Gold Nucleic Acid Gel Stain (Invitrogen) and the results were analyzed with a Typhoon^TM^ Trio + gel scanner (GE Ealthcare). The image was processed by ImageQuant TL8.1 software (image-gathering settings: filter 520 BP 40, Laser Blue 488nm, Sensitivity: normal, PMT 455V).

The red box indicates the part of the image reported in panel (b) of the figure 3 in the manuscript.
